# Connected-Speech Readability as a Biomarker of Psychosis

**DOI:** 10.1192/j.eurpsy.2026.10952

**Published:** 2026-07-31

**Authors:** G. Bubbico, C. Marrangone, G. Pellegrino, D. Sacripante, S. Marino, G. Chiacchiaretta, D. Perpetuini, M. G. Perrucci, M. Pettorruso, G. Martinotti

**Affiliations:** 1 University G. d’Annunzio; 2 Psychiatry Unit, SS Annunziata Hospital, Chieti, Italy, Chieti, Italy

## Abstract

**Introduction:**

Connected speech elicited by picture-description tasks provides a sensitive, non-invasive window into psychopathology. In psychosis, narrative disorganization and linguistic abnormalities can track illness severity and functioning. Readability and lexical indices, complemented by acoustic markers of prosody and spectral shape, may serve as objective, scalable biomarkers for early detection, monitoring, and personalized care.

**Objectives:**

To quantify readability/lexical features of Cookie Theft narratives, test group differences between psychotic disorders (PSI) and healthy controls (HC) on acoustic speech markers.

**Methods:**

Participants were 28 adults (18–65) with DSM-5 psychotic disorders (schizophrenia-spectrum/related), able to complete the Boston Diagnostic Aphasia Examination *Cookie Theft* picture description. Exclusion criteria were major neurological disease, aphasia, intellectual disability, acute substance intoxication, and comorbid major depressive disorder. From verbatim transcripts we computed readability/lexical indices—Gunning Fog, Coleman–Liau, Flesch–Kincaid Grade, Automated Readability Index, SMOG, Flesch Reading Ease, URE score, and lexical density. In a subset with audio recordings (including psychosis and healthy controls), we extracted acoustic features (mean/SD pitch, relative jitter/RAP, RMS energy, spectral centroid/bandwidth/rolloff, zero-crossing rate, MFCCs). Analyses used two-sample t-tests (HC vs PSI) and Pearson correlations with age and illness duration.

**Results:**

Language outputs showed marked complexity: Gunning Fog, Flesch–Kincaid Grade, and SMOG were well above university level, while readability was severely reduced (mean Flesch Reading Ease ≈ −7). Versus HC, PSI produced text with **poorer readability** (Dale–Chall/URE: t=−8.13, p≈0) and **higher lexical density** (t=−2.93, p=0.0056). Acoustically, HC exhibited **higher pitch** (mean: t=5.66, p≈0; variability: t=6.01, p≈0) and **greater spectral brightness/extent** (centroid: t=6.57, p=1.1×10^-7^; bandwidth: t=6.34, p=2.2×10^-7^; rolloff: t=5.82, p=1.1×10^-6^; ZCR: t=2.66, p=0.0116). PSI showed **higher jitter** (t=−2.47, p=0.018), **higher RAP** (t=−2.40, p=0.021) and **greater RMS energy** (t=−3.69, p=0.0007). MFCCs also differentiated groups (e.g., mean MFCC6: t=5.13, p=9.5×10^-6^; mean MFCC8: t=4.13, p=2.0×10^-4^). Exploratorily, **age** correlated with **MFCC2** (r=0.399, p=0.035), while **illness duration** showed trends toward **narrower bandwidth** (r=−0.389, p=0.060) and **higher MFCC1** (r=0.369, p=0.076).

**Conclusions:**

Converging textual and acoustic abnormalities—high lexical density with poor readability, reduced pitch dynamics, narrowed spectra, and greater vocal irregularity—outline a characteristic signature of psychotic speech. If replicated in larger samples, this multimodal profile could function as a practical digital biomarker, complementing clinical scales (e.g., PANSS, BPRS, CGI) for early detection, individualized prognosis, and longitudinal treatment monitoring.

**Disclosure of Interest:**

None Declared

